# Characterization of the complete chloroplast genome of *Chenopodium* sp. (Caryophyllales: Chenopodiaceae)

**DOI:** 10.1080/23802359.2019.1640089

**Published:** 2019-07-16

**Authors:** Luxi Yang, Qiang Li, Gang Zhao

**Affiliations:** a College of Pharmacy and Biological Engineering, Chengdu University, Chengdu, Sichuan, China;; b Key Laboratory of Coarse Cereal Processing, Ministry of Agriculture and Rural Affairs, Chengdu, Sichuan, China;; c National Research and Development Center for Coarse Cereal Processing, Chengdu University, Chengdu, Sichuan, China

**Keywords:** *Chenopodium*, chloroplast genome, phylogenetic analysis

## Abstract

In this study, the complete chloroplast genome of *Chenopodium* sp. were sequenced and annotated. The complete chloroplast genome of *Chenopodium* sp. was composed of circular DNA molecules with a total length of 152,068 bp. The base composition of this chloroplast genome is as follows: A (31.16%), T (31.58%), G (18.27%), and C (18.99%). The chloroplast genome contains 84 protein-coding genes, 8 ribosomal RNA genes (rRNA), and 37 transfer RNA (tRNA) genes. The taxonomic status of the *Chenopodium* sp. chloroplast genome exhibits a closest relationship with Chenopodium quinoa.

*Chenopodium* (Caryophyllales: Chenopodiaceae) is a genus of terrestrial plant with hundreds of species described. *Chenopodium* spp. are widely distributed in the world and have strong environmental adaptabilities (Zou et al. 2017). They are distributed throughout the forests, highlands, and plateaus (Bazile et al. 2016). Some species from the genus have attracted international attention because of their high nutritional value (Abugoch James 2009), such as Quinoa, *Chenopodium formosanum*, and so on. These species contain an excellent balance of essential amino acids, carbohydrates, lipids, minerals, and vitamins (Nowak et al. 2016). Quinoa is considered to be an important crop in the genus *Chenopodium* to improve world food security (Vega-Gálvez et al. 2010). In addition, some bioactive ingredients, such as flavonoids, volatile components, phenolic acid, and phytoecdysteroids, extracted from quinoa and other *Chenopodium* species showed antimicrobial and antioxidant activities (Tang and Tsao 2017). The chloroplast genome of *Chenopodium* sp. reported here will promote further understanding of the genetics, evolution, and taxonomy of this important terrestrial plant.

The specimen (*Chenopodium* sp.) was obtained from Liangshan Yi Autonomous Prefecture, Sichuan, China (102.52 E; 27.38 N) and was stored in Chengdu University (No. Chesp_1). The total genomic DNA of *Chenopodium* sp. was extracted using the CTAB method. A Gel Extraction Kit (Omega Bio-Tek, Norcross, GA, USA) was used to purify the extracted genomic DNA. The purified genomic DNA was stored in the sequencing company (Biomarker Technologies, Beijing, China). Sequencing libraries were constructed with purified DNA following the instructions of NEBNext® Ultra™ II DNA Library Prep Kit (NEB, Beijing, China). Whole genomic sequencing was performed by the Illumina HiSeq 2500 Platform (Illumina, SanDiego, CA, USA). The obtained raw data first passed through a series of quality control steps to obtain clean reads. The chloroplast genome of *Chenopodium* sp. was *de novo* assembled using the SPAdes 3.9.0 software (Bankevich et al. 2012). The MITObim V1.9 software (Oslo, Norway) (Hahn et al. 2013) was used to fill gaps among contigs. The complete chloroplast genome was annotated using the Geseq (Tillich et al. 2017), combined with manual corrections. tRNA genes were predicted using tRNAscan-SE v1.3.1 (Lowe and Chan 2016).

The total length of *Chenopodium* sp. chloroplast genome is 152,068 bp. This chloroplast genome was submitted to GenBank database under accession No. MN031136. The chloroplast genome contains 84 protein-coding genes, 8 ribosomal RNA genes (rRNA), and 37 transfer RNA (tRNA) genes. The base composition of this mitochondrial genome is as follows: A (31.16%), T (31.58%), G (18.27%), and C (18.99%).

To validate the phylogenetic position of *Chenopodium* sp., we constructed the phylogenetic trees of 11 closely related species based on the nucleotide sequences of the chloroplast genome. Bayesian inference (BI) phylogenetic methods were used to construct phylogenetic trees with MrBayes v3.2.6 (Ronquist et al. 2012). Bayesian posterior probabilities (BPP) were calculated to assess node support. As shown in the phylogenetic tree (Figure 1), the taxonomic status of the *Chenopodium* sp. exhibits a closest relationship with *C. quinoa* (Hong et al. 2017).

**Figure 1.**
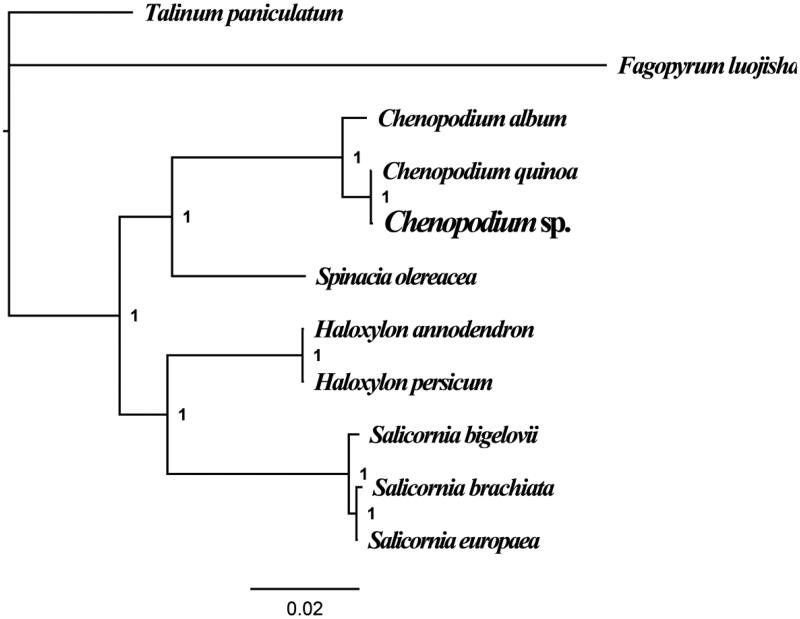
Molecular phylogenies of 11 species based on Bayesian inference analysis of the combined chloroplast gene set. Node support values are Bayesian posterior probabilities (BPP). Chloroplast genome accession numbers used in this phylogeny analysis: *Chenopodium album* (NC_034950); *Chenopodium quinoa* (NC_034949); *Haloxylon ammodendron* (NC_027668); *Haloxylon persicum* (NC_027669); *Salicornia bigelovii* (NC_027226); *Salicornia brachiata* (NC_027224); *Salicornia europaea* (NC_027225); *Spinacia oleracea* (NC_002202); *Fagopyrum luojishanense* (NC_037706); *Talinum paniculatum* (NC_037748).
